# The ratio of urinary sodium and potassium and chronic kidney disease progression: Results from the KoreaN Cohort Study for Outcomes in Patients with Chronic Kidney Disease (KNOW-CKD)

**DOI:** 10.1097/MD.0000000000012820

**Published:** 2018-11-02

**Authors:** Hoseok Koo, Subin Hwang, Tae Hee Kim, Sun Woo Kang, Kook-Hwan Oh, Curie Ahn, Yeong Hoon Kim

**Affiliations:** a Department of Internal Medicine, Inje University Seoul Paik Hospital, Seoul; b Department of Internal Medicine, Inje University Busan Paik Hospital, Busan; c Department of Internal Medicine, Seoul National University School of Medicine, Seoul, Korea.

**Keywords:** chronic kidney disease, urinary potassium, urinary sodium

## Abstract

The Na/K ratio in urine stands for the dietary of sodium and potassium intake in patients with chronic kidney disease remains unclear for the renal progression. We aimed to determine the risk of progression of chronic kidney disease based on the Na/K ratio in a 24-hour urine collection.

We determined the association between the progression of renal disease and 24-hour urinary sodium and potassium (Na/K) ratios in 2238 patients over a 5-year timespan using data obtained from the KoreaN cohort study for Outcomes in patients With Chronic Kidney Disease (KNOW-CKD). Renal events were defined as a 50% decrease in the glomerular filtration rate (GFR) below baseline, or the onset of end-stage renal disease (ESRD). Patients were divided into 4 groups based on the quartile range of the 24-hour urinary sodium and potassium ratio. We analyzed those variables in the 4 groups. Multiple logistic regression analyses were performed using the data of 1001 patients to identify the independent factors associated with renal events.

Age and male sex accounted for the greatest number of patients in the group with the highest values (group 4) of the 24-hour urinary Na/K ratio (≥3.85). There was no difference in the prevalence of hypertension or diabetes mellitus, the ratio of use of antihypertensive drugs, blood pressures, or estimated GFRs. In the group with the highest urinary Na/K ratio, the 24-hour urinary Na concentration mean ± standard deviation was 188.7 ± 70.6 mmol and that of urinary K was 39.9 ± 16.1 mmol. The urinary protein excretion was highest in the group with the highest urinary Na/K ratio. In the logistic regression analysis, the effect on renal events increased with increasing urinary Na/K ratios. After adjusting for other factors, the risk of renal events was 2.48 (95% confidence interval (CI) 1.30–4.90) in group 3, and 3.75 (95% CI: 1.35–11.27) in group 4. In the Kaplan–Meier analysis, the higher the urinary Na/K ratio, the higher the rate of CKD progression.

Based on our analyses, we concluded that the higher the urinary Na/K ratio, the greater the risk of CKD progression.

## 1 Introduction

The excessive intake of salt directly leads to worsening of hypertension.^[1]^ In addition, high salt intake increases the excretion of protein in the urine, resulting in a decrease in renal function.^[2]^ There are some studies that provide evidence for such assertions. In an 11-year observational study of 2196 women with normal renal function, a higher intake of sodium based on questionnaire results showed a faster decline in glomerular filtration rate (GFR).^[3]^ In a study of the effects of ramipril in 500 patients with glomerulonephritis, elevated 24-hour sodium excretions resulted in the development of end-stage renal failure (ESRD). The same result was obtained after adjusting for proteinuria associated with deterioration of renal function.^[4]^ However, some studies have shown that sodium intake is not associated with deterioration of renal function. Approximately 800 nondiabetic patients with nephropathy participated in the Modification of Diet in Renal Disease (MDRD) study. There was no correlation between urinary sodium excretion and ESRD or all-cause mortality.^[5]^ The reason for the difference in the study results was that the potassium intake was not adjusted. Because potassium intake increases the excretion of sodium thereby lowering blood pressure and reducing kidney damage, increased intake of potassium increases the excretion of sodium in the urine and decreases blood pressure.^[6]^ Increased potassium intake also increases Kallikrein secretion, reduces glomerulosclerosis, and inhibits tubular damage.^[7,8]^ The Na/K in urine stands for the dietary of sodium and potassium intake. Therefore, a study suggested that the relationship between the urine sodium/potassium (Na/K) ratio and blood pressure in the general population. When sodium intake was high, blood pressure increased, but if potassium intake was high, there was an increase in excretion of sodium into the urine.^[9]^ In particular the group who had higher sodium intake experienced a greater decrease in blood pressure with higher potassium intake.^[9]^ However, this study was conducted in the general population, and the results of the study in patients with chronic kidney disease were different. In an analysis of 24-hour urine sodium concentrations in 3939 patients with chronic kidney disease, renal outcomes such as the onset of ESRD and 50% decreases in the estimated GFR (eGFR) occurred more frequently with increased excretion of urine sodium and potassium.^[8]^ In this study, the renal outcome was poor when the potassium excretion was high, which was different from that of the general population in general.^[8]^ Therefore, we decided to conduct a study on the progression of renal disease and its relationship to results of 24-hour urine Na/K ratios in patients with chronic kidney disease.

## 2 Materials and methods

### 2.1 Study population and ethical considerations

The KoreaN cohort study for Outcomes in patients With Chronic Kidney Disease (KNOW-CKD) is a prospective cohort study of 2238 patients with nondialysis chronic kidney disease (CKD) stages 1 to 5 who were enrolled between February 2011 and January 2016 in Korea. The detailed design and methods of the KNOW-CKD were previously published.^[10]^ We excluded patients with a previous history of chronic dialysis or kidney transplantation, heart failure (NYHA class 3 or 4) or liver failure (Child–Pugh class 2 or 3), past or current history of malignancy, and current pregnancy. The KNOW-CKD protocol was approved by the institutional review board at each participating hospital, including Seoul National University Hospital, Yonsei University Severance Hospital, Kangbuk Samsung Medical Center, Seoul St. Mary's Hospital, Gil Hospital, Eulji Medical Center, Chonnam National University Hospital, and Busan Paik Hospital.

**Figure 1:**
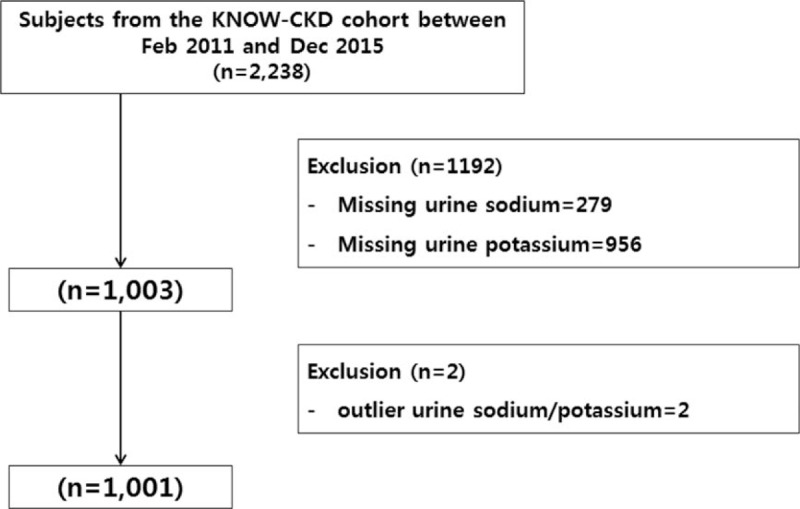
Algorithm for selecting study subjects from the KNOW-CKD cohort. KNOW-CKD = KoreaN Cohort Study for Outcomes in Patients With Chronic Kidney Disease.

Of the 2238 patients, 1192 were excluded comprising 279 patients with missing urine sodium data and 956 with missing urine data. The outliers with respect to the urine Na/K ratio were also excluded (n=2). Ultimately, 1,001 patients were included in the final analysis (Fig. 1).

### 2.2 Data collection and definitions

Demographic data were collected from data registry including: age, sex, systolic blood pressure (SBP), diastolic blood pressure (DBP), body mass index (BMI), medical history of hypertension and diabetes, serum creatinine, 24-hour urine Na/K, total cholesterol, triglyceride (TG), high-density lipoprotein cholesterol (HDL cholesterol), low-density lipoprotein cholesterol (LDL cholesterol), fasting blood sugar (FBS), HbA1c, the presence or absence of metabolic syndrome, and antihypertensive medication history (angiotensin converting enzyme inhibitor, angiotensin receptor blocker, calcium channel blocker), estimated GFR, 24 hours urine protein.

CKD and its stages were defined from the Kidney Disease Improving Global Outcomes (KDIGO) 2012 guidelines.^[11]^ The eGFR was calculated using the 4-variable CKD-EPI equation^[12]^. Metabolic syndrome was defined by the presence of 3 or more of the following: abdominal obesity > 90 cm in men or > 80 cm in women, triglyceride level ≥ 150 mg/dL, or taking medication such as lipid lowering agent, HDL-C level < 40 mg/dL in men or < 50 mg/dL in women, or taking medication, systolic blood pressure ≥ 130 mm Hg and/or diastolic blood pressure ≥ 85 mm Hg, or taking medication, and fasting plasma glucose level ≥ 100 mg/dL, or taking medication. Thresholds for abdominal obesity were defined based on the Asian population.^[13]^ We defined renal events as a 50% decrease of the GFR from baseline and/or the start of renal replacement therapy.

Completeness in the 24-hours urine is determined by the subject's records. And also we defined appropriate urine collection as the ratio of measured 24-hour urinary creatinine to estimated 24-hour urinary creatinine of 0.75 to1.25, and patients with ratio in this range were included. If the ratio over 1.25 or below 0.75, we checked the amount of urine volume and review the urine creatinine and exclude the extreme outliers for urinary creatinine > 3 SD from mean.

### 2.3 Statistical analysis

To determine the characteristics of the subjects, a descriptive analysis was used for continuous variables. For categorical variables, the χ2 test was performed to determine the correlation between different variables. We divided the subjects into quartile according to the 24-hour urine Na/K ratio. To compare the characteristics between the groups, an analysis of variance (ANOVA) was performed. In a multiple logistic regression analysis to determine the incidence of renal events and composite outcomes, we adjusted for the potential confounding factors of age, sex, smoking status, waist circumference, BMI, triglycerides, 24-hour urine protein, and metabolic syndrome. Potassium excretion was affected when the GFR was <15 mL/min/1.73 m^2^,^[14]^ so we performed a subgroup analysis of the patients with GFRs from 15 to 90 mL/min/1.73 m^2^. A Cox proportional hazard model was also used to evaluate the significance of the uric acid level with respect to outcomes. R version 3.1.1. (R Foundation for Statistical Computing, Vienna, Austria) was used for all data analyses. Statistical significance was considered when the *P* value was <.05.

## 3 Results

### 3.1 Baseline characteristics

A total of 1001 patients were categorized 24-hour urinary Na/K ratio into quartile. Across all 4 groups, patients in the highest 24-hour urinary Na/K ratio were group 4 and them who had the lowest were group 1. The oldest patients were found in group 1 and the youngest were found in group 4. The proportion of men was the lowest in group 1 and the highest in group 4. The proportion of smokers was the lowest in group 1 and was higher in groups 2 and 3. The proportion of patients with hypertension was the highest in group 3 and that of diabetes mellitus was the highest in group 1. There was no difference in the ratio of patients with hypertension and diabetes. The use of antihypertensive medication was more than 80% on average, and the use of diuretics was the highest in group 4, but there was no significant difference among the groups. Systolic blood pressure was highest in group 3 and diastolic blood pressure was lowest in group 4. Likewise, statistical significance was not observed, as shown in Table 1.

**Table 1:**
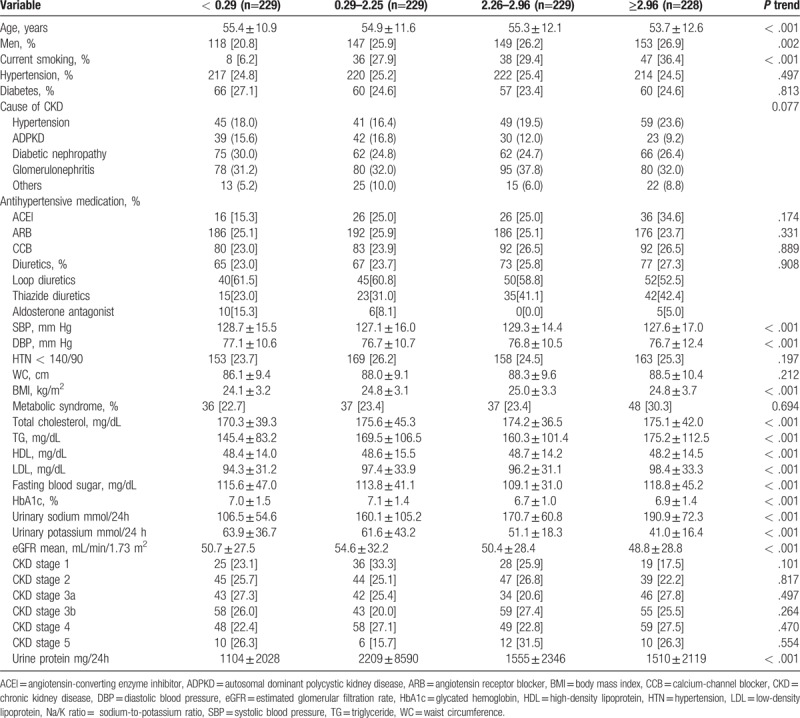
Baseline characteristics of patients according to quartile of 24-hour urinary Na/K ratio.

The mean proportion of hypertensive patients with blood pressures <140/90 mm Hg was 70% overall with no difference between the groups. Waist circumferences were increased in groups 3 and 4. BMI was the highest in group 3. The proportion of patients with metabolic syndrome was lower in groups 1 and 2, but there was no significant difference among the groups. The TG level was the highest in group 4, but the other lipid levels showed no difference among the groups. FBS and glycated hemoglobin were not different among the groups

The average 24-hour urinary sodium was 188.7 mmol in group 4, and the average 24-hour urinary potassium was 39.9 mmol. The average GFR was 47.2 mL/min/1.73 m^2^ in group 4. There was no difference in the proportion of patients with CKD among the groups. Proteinuria was the highest in group 4 (1602 mg).

### 3.2 Effect of the urinary Na/K ratio on renal outcomes

A regression analysis of renal outcomes with respect to age, sex, smoking status, waist circumference, BMI, TG, and 24-hour urine protein was performed. Compared to group 1, the odds ratio of renal outcomes were 2.52 in group 3 (95% confidence interval (CI) 1.39–4.79; *P* = .003) and 2.78 in group 4 (95% CI 1.54 to 5.25; *P < *.001), respectively. The higher the group was, the higher the proportion of renal outcomes that occurred. After adjusting for covariates, the odds ratio of renal outcomes was 2.48 (95% CI 1.30–4.90; *P = *.006) in group 3 and 2.95 (95% CI 1.56–5.81; *P* < .001) in group 4.

Except in cases of GFRs <15 mL/min/1.73 m^2^, which affected the excretion of potassium, the renal outcomes, 50% decrease of the GFR from baseline and/or the start of renal replacement therapy, are as follows: compared to group 1, the risk of renal outcomes was higher in group 3 (OR 2.37; 95% CI 1.15–5.16; *P = *.022) and higher in group 4 (OR 3.27; 95%CI 1.64–6.97; *P < *.001). After adjusting for covariates, the risk of renal outcomes was 2.30 higher in group 3 (OR 2.30; 95% CI 1.06–5.25; *P = *.038), and higher in group 4 (OR 3.48; 95% CI 1.67–7.73; *P = *.001) (Table 2).

**Table 2:**
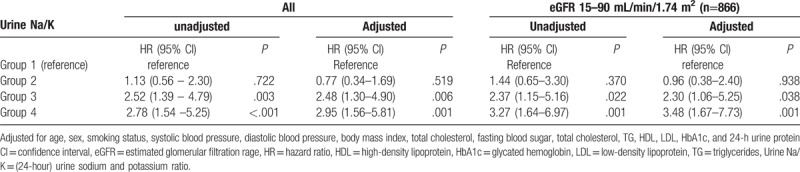
Effect of urinary Na/K ratio on renal outcomes.

**Figure 2:**
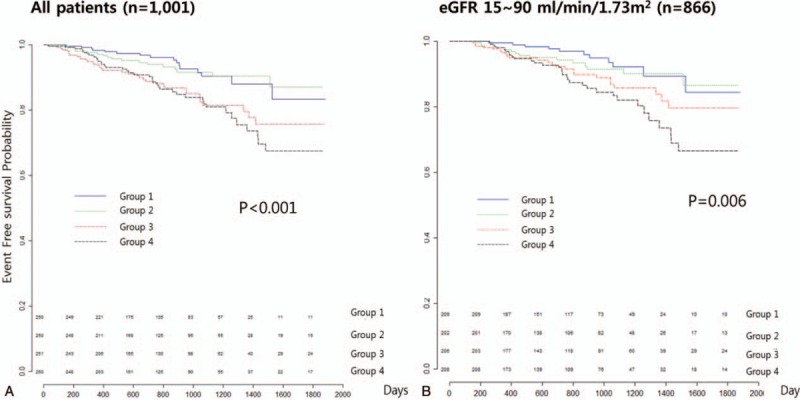
The event-free survival probability of the KM analysis in the urinary Na/K groups. (A) All patients, (B) patients with eGFR 10–90 mL/min/1.73 m^2^. eGFR = estimated glomerular filtration rate, KM = Kaplan–Meier.

In the Kaplan–Meier survival analysis, the event-free survival probability was lower in groups 3 and 4 than in groups 1 and 2 (*P < *.001), and in patients with eGFR 15–90 mL/min/1.73 m^2^, groups 3 and 4 had lower event-free survival probabilities than groups 1 and 2 (*P = *.006) (Fig. 2). In pairwise comparison using Log-rank test, the p-value between group 1 and group 4 was < 0.001, between group 2 and group 4 was 0.049 and showed statistical significance. But there was no statistical significance between group 1 and group 2 and between group 3 and group 4.

### 3.3 The effect of risk factors on renal outcomes compared to urine Na/K ratios

**Figure 3:**
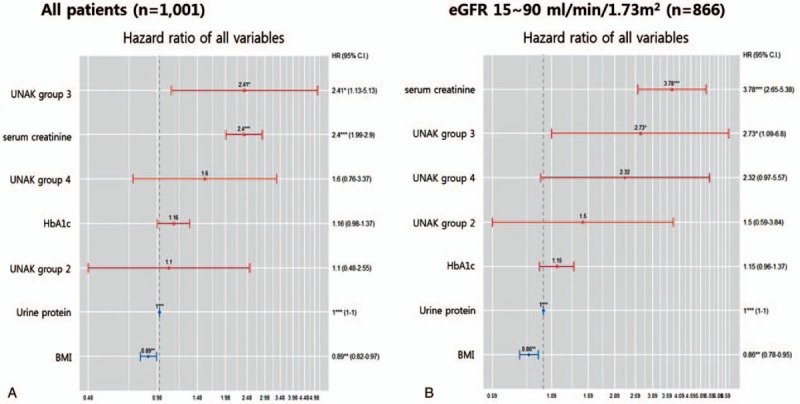
Cox regression analysis of renal outcomes and risk factors. (A) All patients, (B) patients with eGFR 10–90 mL/min/1.73 m^2^. BMI = body mass index, CI = confidence interval, eGFR = estimated glomerular filtration rate, HbA1c = glycated hemoglobin, UNAK = urine Na/K.

We compared other risk factors on renal outcomes. In all patients, the OR was higher in group 3 than the serum creatinine, HbA1c, urine protein, and BMI. In patients with eGFR 15–90 mL/min/1.73 m^2^, serum creatinine had the highest OR, and group 4 had the next highest. The higher the group was, the higher the chance that a renal outcome occurred (Fig. 3).

## 4 Discussion

Sodium intake increases the intravascular blood volume, thus raising blood pressure and consequently increasing pressure in the glomeruli. The overall effect is a decrease in renal function.^[15,16]^ Potassium acts in opposition to sodium, and therefore it increases sodium excretion. Reducing intravascular volume and decreasing blood vessel resistance lowers the blood pressure, which lowers the pressure in the glomerulus and results in stabilization of renal function.^[17]^ Therefore, potassium intake should have a positive effect on renal function. However, as the GFR decreases, potassium excretion becomes less effective and the potassium in the blood increases.^[14]^ In patients with CKD, the results were different in each of the studies that examined the deterioration of renal function using measurements of urine sodium alone or urine potassium alone. The relationship between the ratio of urine sodium and potassium to hypertension has been studied. Systolic and diastolic blood pressures were significantly reduced in 104 patients with moderate essential hypertension who received 30 mmol potassium aspartate daily for 4 weeks. This effect was significant in patients with high urinary Na/K ratios.^[18]^ The relationship between the urinary Na/K ratio and renal function in patients with CKD was not well known. In our study, we found that the greater the excretion of urine sodium, the less the urinary potassium excretion, that is, the greater the 24-hour urine Na/K ratio, the higher the blood pressure and the faster the impairment of renal function.

In addition, if the GFR was <15 mL/min/1.73 m^2^, it may have affected potassium excretion into the urine. In our study, we excluded patients with a GFR of <15 mL/min/1.73 m^2^ and the results were the same.

There is a plethora of research on the recommended daily allowance of sodium. There is a report that the intake of 1.787 to 2.391 g sodium per day lowers cardiovascular disease.^[19]^ Intake of <2.3 g of sodium per day is likely to maintain adequate blood pressure.^[20]^ However, the intake of sodium in Korea based on the 24-hour urine analysis was 3,910 mg per 242 people in Pohang City in 2013.^[21]^ The excretion of potassium is known to be significantly correlated with potassium intake. The potassium intake should be such that the ratio of sodium to potassium is close to 1, that is, the daily potassium intake is 70 to 80 mmol (2.74–3.13 g).^[22]^ Dietary sodium and potassium intervention studies in 1906 Chinese people showed that low sodium and high potassium interventions correlated with blood pressure. People who are sensitive to sodium intake showed more effective blood pressure lowering.^[23]^ Increased potassium intake in people with high salt sensitivity is reported to be more effective for controlling blood pressure. In our study, the urine Na/K ratio in group 3 was more associated with renal impairment. For people at the same level as group 2, the average daily intake of sodium should be <160.1 ± 105.2 mmol/day, and potassium should be consumed in excess of 61.6 ± 42.2 mmol/day to decrease the negative effects on renal function. Sodium should be consumed in amounts < 3680 ± 2,418 mg, and potassium should be consumed in amounts >2,407 ± 1649 mg per day. This includes the WHO recommended Na/K 1:1 intake. When sodium is converted to salt, it should be consumed at <9.2 ± 6.0 g, and potassium should be consumed at the recommended daily dose of 3,000 mg. Additionally, if GFR is decreased, potassium should be consumed with caution.

After the use of a diuretic, the sodium reabsorption in the thick ascending limb is decreased, urinary sodium is increased, and urinary potassium excretion also increases. If the use of diuretics is not confirmed, the results of the study may be limited. In our study, there was no difference in diuretic use among the 4 groups in the 24-hour urine Na/K ratio. There was no significant difference in the GFR that affected the excretion of potassium among the groups. Also, there was no difference in the percentage of CKD among the groups. All the factors that might affect urinary sodium and potassium excretion were adjusted. After that, it was confirmed that if the urine sodium was high and the potassium was low, the renal function would deteriorate quickly.

Among the factors that could affect the deterioration of renal function, age,^[24]^ sex,^[25]^ smoking status,^[26]^ blood pressure,^[27]^ BMI,^[28]^ dyslipidemia,^[29]^ hyperglycemia,^[30]^ and proteinuria^[31]^ were different among the urinary Na/K ratio groups. In a regression analysis of renal outcomes, BMI, HbA1c, serum creatinine and proteinuria, and 24-hour urinary Na/K ratios were significant. In the Cox analysis, groups 3 and 4 showed more renal outcome than the other groups. The level of the ratio of urinary sodium and potassium in group 3 and 4 was also found to have exacerbated the worsening of renal function compared to the other factors exclude the eGFR below 15 mL/min/1.73 m^2^.

The limitations of this study include the relatively short observation period and the fact that other factors affecting renal function deterioration were not properly evaluated. A re-analysis should be performed with a longer observation period. The collection of the 24-hour urine was not well evaluated, and there may have been an error in its interpretation. The amount of urine proteinuria may have affected the degree of deterioration of renal function. There is a study that divided urine proteinuria by 1 g. ^[32]^ We did not analyze it separately, so an accurate assessment of the impact of proteinuria could not be made. However, our study was conducted by collecting 24-hour urine specimens for more than 1000 patients with CKD.

Unlike previous studies, the 24-hour urine Na/K ratio was analyzed. The benefit of this method was that both effect of sodium and potassium was considered. Also the GFR, which affects potassium excretion, was excluded, except in cases where it was <15 mL/min/1.73 m^2^. In our study, we found that low sodium intake and high potassium intake could delay the deterioration of renal function. However, additional analysis is needed after evaluating the 1:1 ratio of urinary sodium and potassium before and after accurate urine volume assessments, and increasing the observation period.

## 5 Conclusion

In patients with CKD, the higher the ratio of urine sodium and potassium over a 24-hour period, the faster the deterioration of renal function.

## Author contributions

**Conceptualization:** Hoseok Koo.

**Data curation:** Tae Hee Kim.

**Formal analysis:** Hoseok Koo, Subin Hwang, Tae Hee Kim.

**Funding acquisition:** Curie Ahn.

**Investigation:** Kook-Hwan Oh, Curie Ahn, Yeong Hoon Kim.

**Methodology:** Sun Woo Kang.

**Supervision:** Kook-Hwan Oh, Curie Ahn, Yeong Hoon Kim.

**Writing – original draft:** Hoseok Koo.

**Writing – review & editing:** Tae Hee Kim.
